# Evaluation of the Effect of Preharvest Melatonin Spraying on Fruit Quality of ‘Yuluxiang’ Pear Based on Principal Component Analysis

**DOI:** 10.3390/foods12183507

**Published:** 2023-09-21

**Authors:** Liangliang Zhao, Shuai Yan, Yufei Wang, Gongxun Xu, Deying Zhao

**Affiliations:** Research Institute of Pomology, Chinese Academy of Agricultural Sciences, Xingcheng 125100, China; 82101205148@caas.cn (L.Z.); yanshuai@caas.cn (S.Y.); 82101215193@caas.cn (Y.W.); gongxunxu1993@stu.syau.edu.cn (G.X.)

**Keywords:** *Pyrus bretschneideri*, exogenous melatonin, fruit quality, soluble sugar

## Abstract

Melatonin (MT), an indoleamine compound, has a pleiotropic effect on plant growth and development and can regulate the quality of tree fruit. Systematic research on the effect of preharvest MT spraying on pear fruit quality and technical solutions for MT application to regulate pear fruit quality are still lacking. Thus, here we aimed to evaluate the effects of different spraying times, concentrations, and exogenous MT application times on ‘Yuluxiang’ pear fruit quality. Our results showed that the single fruit weight and vertical and horizontal diameters of pear fruit sprayed with MT twice at 30 and 90 d after full bloom were the largest, and the red and green values of the treatment were the highest. MT-treated pears had higher contents of total soluble solids, soluble sugar, sucrose, sorbitol, fructose, and glucose and lower contents of titratable acid, malic acid, and citric acid. Moreover, exogenous MT treatment increased the pear peel strength. Based on the principal component analysis of 10 fruit quality indices, the suitable periods for MT spraying on ‘Yuluxiang’ pears were 30 and 90 d after full bloom, the suitable concentration was 100 μmol/L, and the suitable number of times was two. This study provides a theoretical reference for optimizing MT application and improving pear fruit quality.

## 1. Introduction

Pears belong to the family Rosaceae and are one of the most important fruit commodities worldwide. Pears are widely planted in six continents, mainly in China, the United States, Italy, Argentina, and Spain [1]. In recent years, the demand for high-quality pears has increased. To meet the expectations of consumers, it is important to improve the quality of pear fruit [2]. ‘Yuluxiang’ pears are a high-quality, medium-maturing, crisp, juicy, and high-sugar-content pear variety. It is one of the main pear varieties in northern China and is deeply loved by consumers. However, in some producing areas, ‘Yuluxiang’ pear fruit is small, the color is poor, the fruit sugar content is low, and the economic benefits are greatly reduced. Therefore, it is of great significance to study methods to improve the fruit quality of ’Yuluxiang’ pears. Chemical regulation technology changes the endogenous hormone system of plants by applying plant growth regulators to regulate plant growth and development, thereby changing the direction and degree expected by people [3]. This technology has the advantages of rapid effects, low dosage, safety, simple operation, and ease of promotion. Chemical regulations can replace many traditional cultivation and management measures in agricultural production, simplify the agricultural production steps, and become a leading technology for future agricultural development. Plant growth regulators are the foundation of chemical regulation technologies. Melatonin (MT), or N-acetyl-5-methoxytryptamine, a plant growth regulator, is an indole substance synthesized from tryptophan in a matrix. As a pleiotropic factor, it has several biological functions in plants. It is involved in physiological processes such as photosynthesis [4], seed germination [5], fruit enlargement [6], root development [7], and osmotic regulation [8] and plays an important role in plant resistance to environmental stress [9,10].

Higher plants have an MT biosynthesis pathway similar to that in animals [11]. MT formation in plants begins with tryptophan and involves the catalytic reactions of tryptophan decarboxylase (TDC), tryptamine-5-hydroxylase (T5H), tryptophan hydroxylase, serotonin N-acetyltransferase (SNAT), N-acetyl serotonin methyltransferase (ASMT), and caffeic acid O-methyltransferase (COMT). At the beginning of the synthesis, tryptophan is decarboxylated to form tryptamine under the catalysis of TDC and then hydroxylated to serotonin (5-hydroxytryptamine) under the catalysis of T5H. Subsequently, 5-hydroxytryptamine is methylated to 5-methoxytryptamine under the catalysis of ASMT and COMT, and MT is formed via the acetylation of SNAT. In another pathway, serotonin can also be acetylated to N-acetyl serotonin under the catalysis of SNAT and then methylated to form MT [12]. With significant progress in the study of the plant MT biosynthetic pathway, researchers have begun to explore its degradation metabolism. Currently, the research on MT catabolism is in its infancy. There are two known plant MT metabolic pathways: nonenzymatic and enzymatic conversion pathways. The nonenzymatic degradation of free radicals or other reactive oxygen species and the direct photocatalytic reaction to ultraviolet light are similar to those in animals [13].

MT promotes fruit development and ripening. Verde et al. [14] studied the role of MT during the growth and ripening of apples. They found that MT increased fruit size and weight. Tomato seeds were soaked with 100 μmol/L MT before germination to improve fruit quality and yield [15]; it was found that preharvest spraying of MT could regulate the accumulation of primary and secondary metabolites during tomato fruit ripening. MT application improved the nutritional and flavor quality of tomato fruit by regulating the accumulation of primary and secondary metabolites during ripening [16]. Treatment of young grape fruit with 100 mg/L MT could promote fruit enlargement and increase fruit weight by 6.6%. Simultaneously, it reduced under-ripening and over-ripening of the fruit and improved its synchronization [17]. Exogenous MT treatment altered carbohydrate metabolism, polyphenol metabolism, ethylene biosynthesis, and signal transduction in grapes, and this promoted fruit ripening [18]. Exogenous antioxidants promoted fruit ripening by increasing ethylene generation through the upregulation of 1-aminocyclopropane-1-carboxylic acid synthase expression [19]. MT plays an important role in plant resistance to environmental stress. MT levels increased when strawberries were subjected to salt stress, and foliar application of MT could promote this increase and increase fruit yield and quality parameters [20]. Moreover, foliar application of 100 μmol/L exogenous MT could improve the developmental traits of tomato fruit under acid rain stress, increasing the total soluble solids, soluble sugar, and soluble protein content and decreasing the pH values in tomato fruit [21].

MT regulates sugar metabolism in fruit. Sugars are important components of fruit, and their composition and content are important factors in determining fruit quality and flavor. Sugar accumulation is closely related to fruit ripening [22]. Sugar is synthesized via photosynthesis in leaves and accumulates in fruit through a series of physiological processes [23]. Metabolism and transport during fruit development determine the composition and concentration of sugars during fruit ripening [24]. Soluble sugars in fruit mainly include sucrose, fructose, glucose, and sorbitol. Exogenous MT could increase soluble sugar content in grapes by increasing the activity of sucrose phosphate synthase, particularly sucrose [25]. Liu et al. [6] found that exogenous MT treatment improved the photosynthetic efficiency of pear trees, increased the soluble sugar content in the fruit, reduced the expression level and enzyme activity of the *Pyrus bretschneideri* (Pb) sucrose invertase gene Pbinvertase1/2, increased the expression level and enzyme activity of the sucrose phosphate synthase (SPS) gene PbSPS1/2/3, and increased the sugar content of the fruit.

MT affects organic acid content in fruit. The composition and content of organic acids in fruit are important factors affecting fruit acidity, which is one of the decisive factors affecting fruit sensory quality and consumer acceptance [26]. Organic acids in fruit include malic, citric, quinic, shikimic, oxalic, succinic, fumaric, tartaric, acetic, chlorogenic, and ascorbic acids [27]. The acidity of citric acid is higher than that of malic acid, and the bitterness of fruit is primarily caused by quinic and shikimic acids. After the fruit begins to develop, organic acids gradually accumulate in the fruit. During the fruit-ripening stage with glycolysis, the tricarboxylic acid cycle, and glycogen heterogenesis, organic acids are gradually consumed, so their content gradually decreases [28]. During fruit ripening, the amount of organic acids transported from leaves to fruit is reduced, which reduces the organic acid content in fruit [29]. MT may reduce the conversion rate of phosphoenolpyruvate (PEP) to malic acid by reducing the level of PEP carboxylase kinase while increasing the level of malate dehydrogenase and accelerating the conversion of malic acid to oxaloacetic acid, resulting in a decrease in fruit acidity. In addition to the growth regulators that can significantly affect the sugar and acid content of fruit, factors like light [30], temperature [31], moisture [32], soil fertility [33], and others have the same effect.

In this study, we aimed to develop a method to improve the quality of pear fruit by spraying MT. Currently, MT is primarily used for postharvest fruit treatment. Systematic research on the effect of preharvest MT spraying on pear fruit quality and technical solutions for MT application to regulate pear fruit quality are lacking. In view of this, this study aimed to systematically study the effect of exogenous MT on the quality of ‘Yuluxiang’ pear fruit to explore the regulation of MT on the appearance quality, internal quality, and texture quality of the fruit. Based on the principal component analysis, an appropriate MT-spraying scheme was selected to provide theoretical guidance for the improvement of pear fruit quality. The findings in this work are expected to provide new strategies for improving pear production efficiency.

## 2. Materials and Methods

### 2.1. Materials

The test pear variety was ‘Yuluxiang’ (*Pyrus bretschneideri*). The materials were procured from the pear cultivation demonstration base of the Institute of Pomology, Chinese Academy of Agricultural Sciences (Xingcheng city of Liaoning province, 40°37′48′′ N, 120°40′48′′ E). A total of 117 healthy, eight-year-old ‘Yuluxiang’ pear trees with basically the same growth were selected for the experiment. Soil available nitrogen, potassium, phosphorus, organic matter, and pH were 159.25 mg/kg, 239.53 mg/kg, 38.80 mg/kg, 15.81 g/kg, and 7.01, respectively. The row spacing of ‘Yuluxiang’ pear trees was 1 m × 3.5 m, and the rootstock was *Pyrus betulaefolia*. Consistent weed control, irrigation, fertilization, and disease and pest control were performed across the whole orchard to ensure the normal growth of fruit trees. MT used in the experiment was produced by Beijing Soleibao Technology Co., Ltd.: Beijing, China; Tween-80 by Tianjin Tianli Chemical Pharmaceutical Co., Ltd.: Tianjin, China; and anhydrous ethanol by the State Pharmaceutical Group Chemical Reagents Co., Ltd.: Shanghai, China.

### 2.2. Field Experiments

This experiment was conducted at the pear cultivation demonstration base of the Institute of Pomology, Chinese Academy of Agricultural Sciences (Xingcheng, Liaoning, China), from May to December 2022. Xingcheng City, Liaoning province, is located in a warm-temperate to subtemperate climate zone. The four seasons are distinct, the climate is mild, and the light is sufficient. The annual average temperature is 9.2 °C, the annual average precipitation is about 600 mm, and the frost-free period is 175 d. Twelve treatments were designed for this experiment. The concentration and duration of MT spraying were obtained from the relevant literature [6]. The specific treatments are listed in Table 1.

Water was sprayed as control or check (CK). Each treatment was divided into three plots, with three trees in each plot, a random distribution, and one–two protection trees set between different plots. To prepare the solution, the same amount of anhydrous ethanol (0.1%) was added to promote dissolution, and Tween-80 (0.1%) was added after dissolution. The whole-plant-spraying method was employed, with each spraying based on the condensation of water droplets on the leaf and fruit surfaces. Each treatment consumed about 10 L of MT solution. Leaves and fruit from each treatment were collected on 9 June, 25 June, 11 July, 26 July, 11 August, 23 August, 30 August, 7 September, 13 September, and 20 September for index determination. Sampling was repeated for both the single and mixed plants. Four fruits were picked from four directions (east, west, south, and north) of each tree, with a total of 10 leaves in the middle of the new shoots.

### 2.3. Appearance Quality

Single fruit weight was measured using an ACS-30 A electronic balance (Shanghai Yousheng Weighing Apparatus Co., Ltd., Shanghai, China), the vertical and horizontal diameters of the fruit were measured using a DL91150 electronic digital vernier caliper (Deli Group Co., Ltd., Ningbo, China), and the color difference of fruit was measured using a 3nh precision colorimeter (Shenzhen Sanli Instrument Co., Ltd., Shenzhen, China). Each treatment was repeated 6–10 times.

### 2.4. Internal Quality

The fruits were homogenized using a homogenizer, and the total soluble solids content of the homogenate was determined using a handheld refractometer (PAL-1 type, ATAGO, Tokyo, Japan). The titratable acid contents of the homogenates were determined using an automatic potentiometric titrator (905 type, Switzerland) with 1% sodium hydroxide. 

Soluble sugar content was determined using anthrone colorimetry. About 0.1 g of dried plant sample was taken to record the sample amounts. It was put into a 25 mL test tube with a stopper, 5–10 mL distilled water was added, it was sealed with plastic film, and it was extracted in a boiling water bath for 30 min; the supernatant was filtered into a 25 mL volumetric flask. After sealing with 5–10 mL of distilled water and boiling in a water bath for 30 min, all samples were filtered into a volumetric flask, and the test tube and residue were washed repeatedly to scale. After reaching a constant volume, the extracts were diluted 20 times. One milliliter of the diluted extract was added to a dry test tube, and anthrone ethyl acetate reagent (0.5 mL) was added, followed by the addition of 5 mL concentrated sulfuric acid, which was slowly added, shaken well, and immersed in boiling water. The tube mouth was covered to prevent evaporation, and the boiling water bath was removed for 10 min. After cooling to room temperature under running water and standing for 10 min, the absorbance was quickly determined using an ultraviolet (UV) UV-2550 spectrophotometer (Shimadzu, Tokyo, Japan) at 620 nm (completed within two hours). The glucose content was calculated using a standard curve based on the absorbance value.

Ultraperformance liquid chromatography (UPLC)-photodiode array detection (PDA)-mass spectrometry (MS)/MS-electrospray ionization (ESI) (Waters, Milford, MA, USA) was used to determine the sugar and acid contents, with each treatment repeated three times. 

The extraction and purification of sugar components was as follows: An amount of 5.0 g fruit after freezing and grinding was weighed, fixed in 50 mL methanol, shaken, placed in an ultrasonic wave for five minutes, and then centrifuged at 10,000 r/min for five minutes. The extract (2 mL) was evaporated out of the organic phase on a rotary evaporator at 40 °C, and the remaining water phase was poured into an activated solid-phase extraction column (Waters Oasis^®^ HLB, 200 mg/6 CC). The solid-phase extraction column was washed with 5 mL water each time, collected, and washed three times; the eluents were combined (constant volume 20 mL), diluted by a certain multiple according to need, and passed through a 0.22 μm organic-phase filter membrane, followed by UPLC-PDA-MS/MS-ESI sample loading for instrumental analysis. 

The UPLC conditions of sugar components were assessed as follows: A Waters ACQUITY UPLC ethylene bridged hybrid (BEH) Amide column was used. 

The MS/MS conditions of sugar components were as follows: an ESI ion source, negative ion detection mode, multiple-reaction-monitoring (MRM) mode, an ion source temperature of 150 °C, a desolvation gas temperature of 500 °C, a desolvation gas flow rate of 700 L/h, a cone gas flow rate of 50 L/h, and a collision gas (high-purity argon) flow rate of 0.13 mL/min. 

The recovery rate of sugar components was as follows: Standard solutions of sorbitol, glucose, fructose, and sucrose at known concentrations were added to the sample, and the chromatographic conditions remained unchanged. The external standard addition method was repeated thrice to obtain the average recovery rate of each sugar component. The recovery rates of the four sugars in this experiment were 92.61%, 88.51%, 88.49%, and 90.57%, respectively. The unit of sugar determined was mg/100 mg (%).

The extraction and purification of acid components were as follows: Frozen (–80 °C) and ground fruit (5.0 g) were weighed and diluted to 50 mL with water. After shaking well, the solution was placed in an ultrasonic bath for five minutes and centrifuged at 10,000 r/min centrifuge for another five minutes. The extract (5 mL) was poured into an activated solid-phase extraction column (Waters Oasis^®^ HLB, 200 mg/6 CC), and the solid-phase extraction column was washed with 2 mL water each time. The column was then collected and washed twice. The eluents were combined, and the volume was 10 mL. According to need, it was diluted by a certain multiple and passed through a 0.22 μm organic-phase filter membrane, and the sample was loaded to UPLC-PDA-MS/MS-ESI for instrumental analysis. 

The UPLC conditions of acid components were as follows: A Waters Atlantis^TM^ Premier BEH C18 Anion Exchange chromatographic column was used. 

The MS/MS conditions of acid components were as follows: an ESI ion source, negative ion detection mode, MRM mode, an ion source temperature of 150 °C, a desolvation gas temperature of 500 °C, a desolvation gas flow rate of 700 L/h, a cone gas flow rate of 50 L/h, and a collision gas (high-purity argon) flow rate of 0.13 mL/min.

The recovery rate of acid components was as follows: Standard solutions of quinic acid, malic acid, shikimic acid, and citric acid at known concentrations were added to the sample. The chromatographic conditions remained unchanged. The external standard addition method was repeated thrice to obtain the average recovery rate of each sugar component. The recovery rates of the four acids in this experiment were 89.01%, 90.00%, 89.41%, and 91.05%, respectively. The unit of acid determined was mg/g.

### 2.5. Texture Quality

Peel rupture distance, peel puncture strength, pulp puncture strength, and pulp fineness were measured using a texture analyzer (TA) (TA.HD.Plus, Surrey, UK). The common fruit puncture procedure was selected for the determination, and a P/2 probe was used. The instrument parameters were as follows: a preloading speed of 3 mm/s, a pressing speed of 1 mm/s, an upward speed of 3 mm/s after pressing, distance mode, a puncture distance of 3 mm, and a trigger value of 5 g. The fruit hardness index (with skin) was tested near the fruit equator; 10 fruit samples were tested for each treatment, and each fruit was tested twice. Peel strength = peel puncture strength/probe area; pulp hardness = pulp puncture strength/probe area. 

The determination method of stone cell content in pear pulp was based on the NY/T 1388-2007 ‘Determination of stone cell content in pear pulp by gravimetric method’.

### 2.6. Statistical Analysis

Microsoft Excel (V.2016, Washington, DC, USA) was used to calculate the measured values and plot the chart, Statistical Product and Service Solutions (SPSS) (V.20.0, New York, NY, USA) was used to analyze the results, multiple comparisons were made using analysis of variance, and Duncan’s multirange test (*p* ≤ 0.05) was used to plot the chart. A principal component analysis (PCA) was performed using the dimensionality reduction factor in SPSS 26.0. Lowercase letters indicate statistically significant differences for the same storage period. Data are presented as mean ± standard error.

## 3. Results

### 3.1. Effects of Exogenous MT on Appearance Quality of Pear Fruit

**Figure 1:**
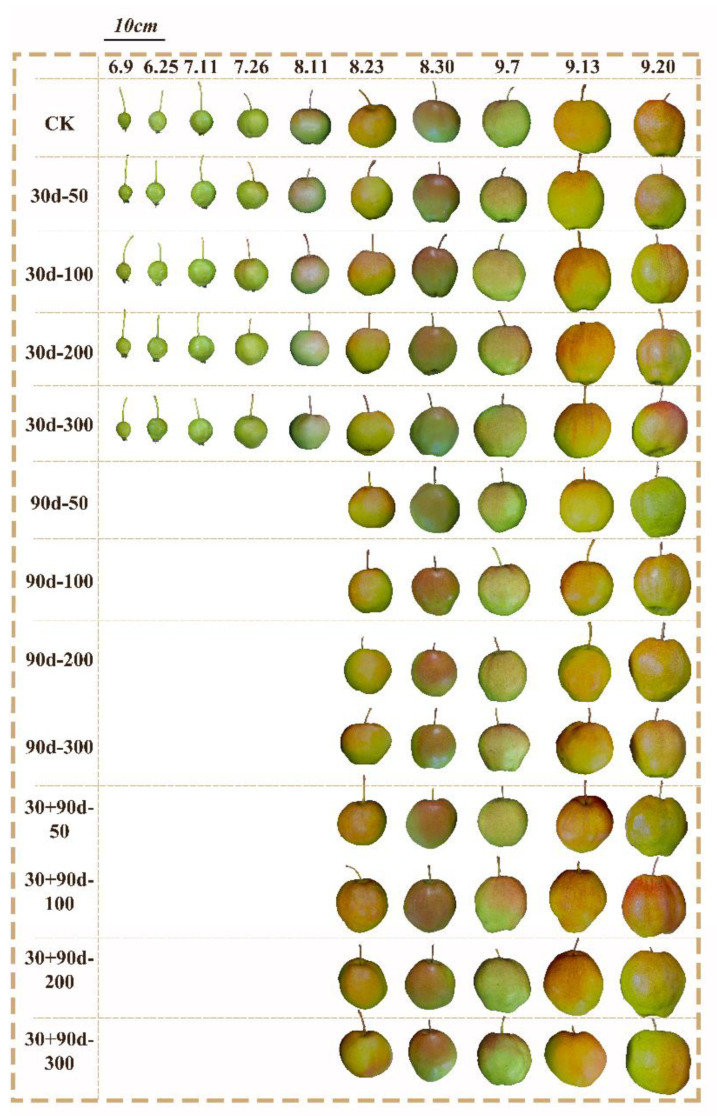
Effects of exogenous MT on appearance quality of pear fruit.

**Figure 2:**
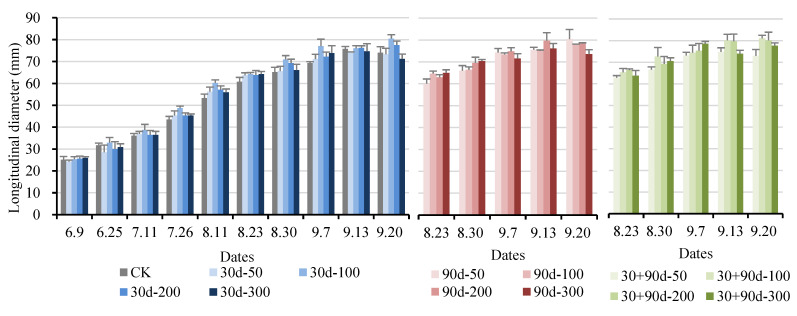
Changes in fruit longitudinal diameter for different MT-spraying schemes.

**Figure 3:**
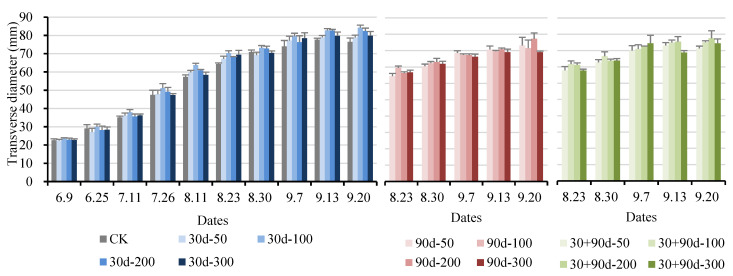
Changes in fruit transverse diameter for different MT-spraying schemes.

Figure 1 shows the fruit appearance of different treatments in different periods. As shown in Figure 2 and Figure 3, the longitudinal and transverse diameters of ‘Yuluxiang’ pears increased to different degrees after different MT-spraying treatments. From 9 June to 11 August, the vertical and horizontal diameters of the 30 d-50, 30 d-100, 30 d-200, and 30 d-300 treatments increased rapidly compared with those of CK, and the overall performance was 30 d-100 > 30 d-200 > 30 d-50 > 30 d-300 > CK. At the fruit maturity stage (20 September), the longitudinal diameter of the 30 + 90 d-100 treatment was the largest at 81.07 mm, 9.5% higher than that of CK. The transverse diameter of the 30 + 90 d-200 treatment was the largest, reaching 87.59 mm, 14.5% higher than that of CK.

**Figure 4:**
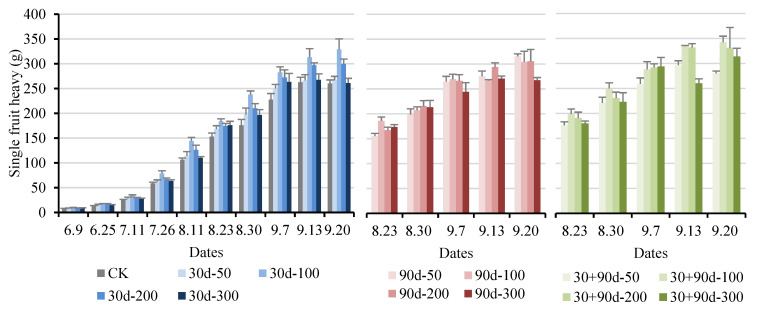
Changes in single fruit weight for different MT-spraying schemes.

Figure 4 shows that the single fruit weight of ‘Yuluxiang’ pears increased by different degrees under different MT-spraying schemes compared with the control. From 9 June to 11 August, the trend of the single fruit weight of pears was like that of the vertical and horizontal diameters. At the fruit maturity stage, the single fruit weight of the 30 + 90 d-100 treatment was the highest at 342.36 g, 31.6% higher than that of CK. The single fruit weight of the 30 d-100 treatment was also at a high level during the entire developmental period, reaching 328.88 g at maturity, 26.4% higher than that of CK.

**Figure 5:**
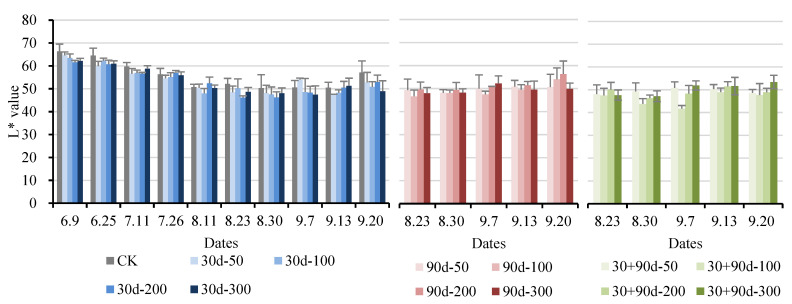
Changes in L* value of fruit for different MT-spraying schemes.

The L* value indicates brightness: a positive value indicates bright color, while a negative value indicates darkness. Figure 5 depicts that the L* value of ‘Yuluxiang’ pear fruit showed a trend of decreasing first and then increasing. The L* value of pears decreased after exogenous MT treatment. The L* value of CK was high during the entire development process, whereas the L* value for the 30 + 90 d-100 treatment was low at the later stage. At maturity, the L* value for the 30 + 90 d-100 treatment was 47.66, 16.5% lower than that of CK, indicating that exogenous MT reduced the brightness of pear fruit.

**Figure 6:**
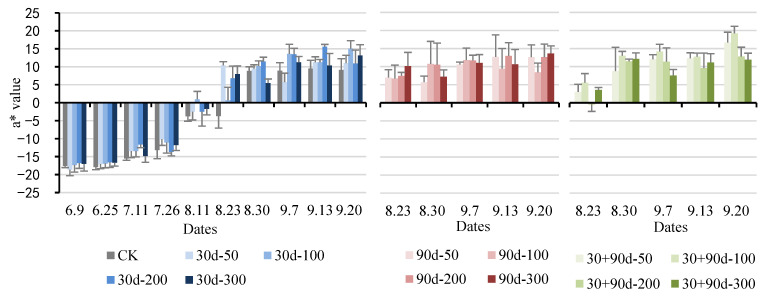
Changes in a* value of fruit for different MT-spraying schemes.

The a* value represents the red and green colors: the value is positive for redness and negative for greenness. Figure 6 shows that the a* value of ‘Yuluxiang’ pear fruit demonstrated a rising trend. The a* values for the different MT-spraying schemes were higher than that of CK to varying degrees. The a* value of CK was lower during the entire development process, whereas that for the 30 + 90 d-100 treatment was higher in the late stage of fruit development. On 23 August, the a* value for the MT treatments were positive, whereas the a* value of CK was still negative. At the mature stage, the a* value for the 30 + 90 d-100 treatment was the highest at 19.22, 110.1% higher than that of CK, indicating that exogenous MT treatment promoted pear fruit redness. Among these, the 30 + 90 d-100 treatment showed the best effect.

**Figure 7:**
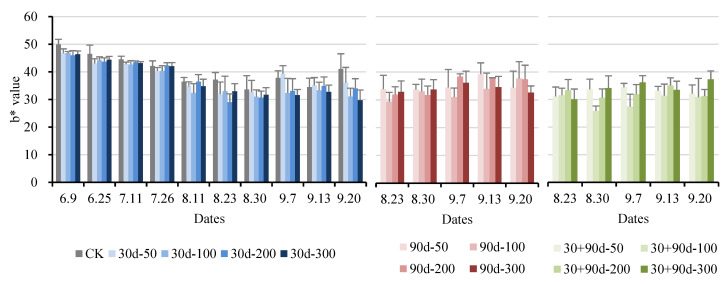
Changes in fruit b* value for different MT-spraying schemes.

The b* value represents the yellow–blue value: a positive b* indicates a yellowish color, and a negative indicates a blue color. Figure 7 shows that ‘Yuluxiang’ pears were always yellow throughout the development process. The b* values of pears after exogenous MT treatment showed a decreasing trend. The b* value of CK was high throughout the developmental process. The 30 + 90 d-100 treatment was at a low level during the later stages. At maturity, the b* value for the 30 + 90 d-100 treatment was 30.90, 24.8% lower than that of CK, indicating that exogenous MT treatment inhibited pear fruit yellow color.

**Figure 8:**
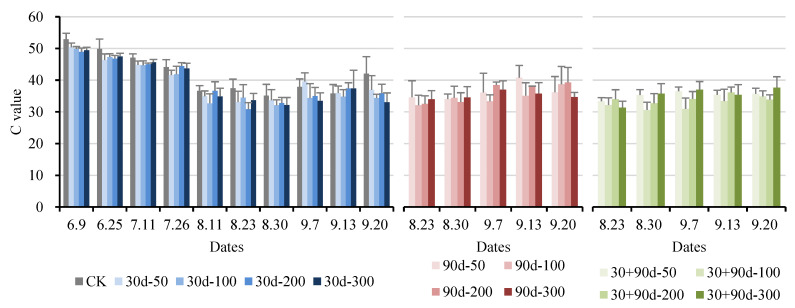
Changes in fruit C value for different MT-spraying schemes.

The C value represents the fruit color saturation. The higher the C value, the higher the color purity of the fruit and a more single color composition. The lower the C value, the more complex the color composition of the fruit, and the lower the color purity. As shown in Figure 8, the C values of the pears decreased after exogenous MT treatment. The C value of CK was high throughout the developmental process. The C value for the 30 + 90 d-100 treatment was lower at the later stages. At the mature stage, the C value of the 30 + 90 d-100 treatment was 34.87, 17.2% lower than that of CK, indicating that exogenous MT treatment reduced the color saturation of pear fruit.

**Figure 9:**
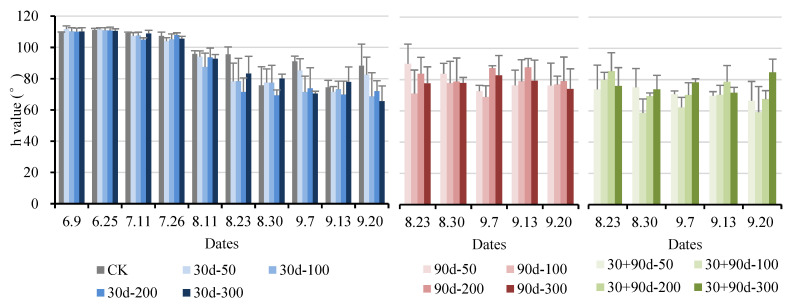
Changes in fruit h value for different MT-spraying schemes.

The hue angle, h, represents the color difference in the peel. The h values of 270°, 180°, 90°, and 0° are expressed as blue, green, yellow, and red, respectively. The smaller the h value, the greater the color change in the peel. As shown in Figure 9, the h value of pears showed a decreasing trend after exogenous MT treatment, and the h value of CK was always high. At the maturity stage, the h value for the 30 + 90 d-100 treatment was the smallest, 33.0% lower than that of CK, indicating that exogenous MT treatment promoted the redness of pear fruit, and the effect of the 30 + 90 d-100 treatment was the best.

### 3.2. Effects of Exogenous MT on Internal Quality of Pear Fruit

**Figure 10:**
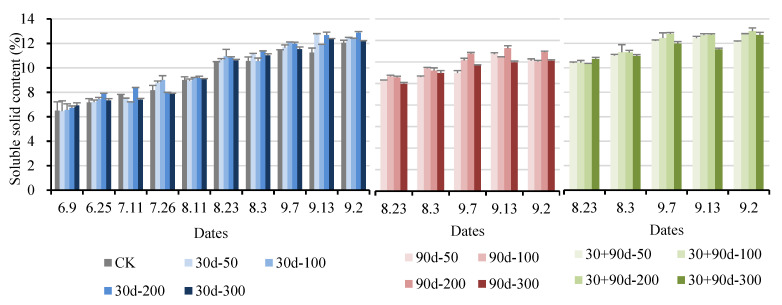
Changes in fruit soluble solids content for different MT-spraying schemes.

As shown in Figure 10, spraying MT affected the soluble solid content of pear fruit during development. From 9 June to 11 August, spraying different MT concentrations increased the soluble solid content of the pear fruit. After 23 August, the soluble solids content for the 90 d-200, 30 + 90 d-100, and 30 + 90 d-200 treatments increased rapidly. On 13 September, the soluble solids content for the 90 d-200 treatment reached the highest level at 13.3%, 4.5% higher than that of CK. The soluble solids contents for the 30 + 90 d-100 and 30 + 90 d-200 treatments were the highest at 12.7% and 13.0%, as well as 5.6% and 7.9%, respectively, higher than that for CK.

**Figure 11:**
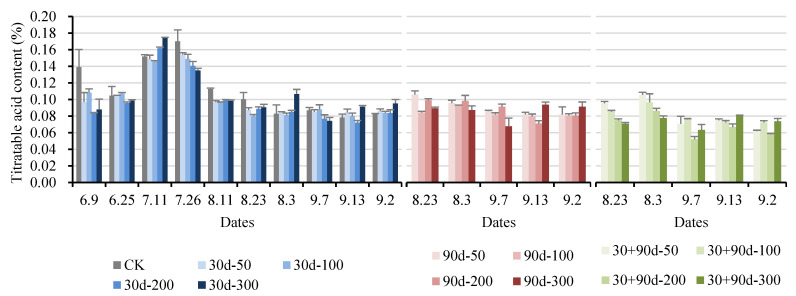
Changes in fruit titratable acid content for different MT-spraying schemes.

The titratable acid content of ‘Yuluxiang’ pears increased first and then decreased (Figure 11). After 26 July, the titratable acid content for 30 d-100 decreased rapidly, reaching 0.080% on 23 August, 20.0% lower than that of CK, and then stabilized in the range of 0.080–0.088%. After 7 September, the titratable acid content for the four 30 + 90 d treatments decreased significantly. At maturity, the titratable acid content for 30 + 90 d-200 was the lowest at 0.058%, 30.1% lower than that of CK. The titratable acid contents for the 30 d-300 and 90 d-300 treatments were significantly higher than that of CK.

**Figure 12:**
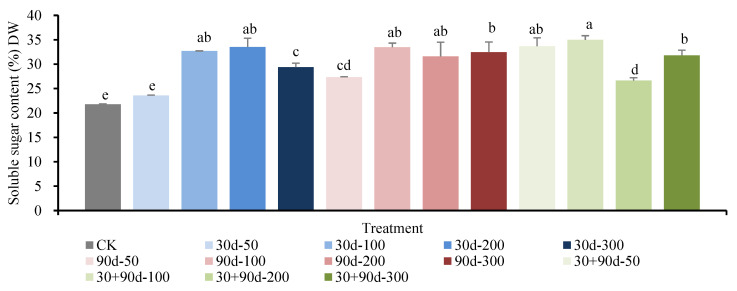
Soluble sugar content of fruit in mature stage for different MT-spraying schemes. Different lowercase letters indicate that the content of soluble sugar was significantly different in different treatments (*p* < 0.05).

As shown in Figure 12, different MT-spraying treatments increased the soluble sugar content of mature pear fruit to varying degrees. Except for the 30 d-50 treatment, the sugar contents for all other treatments were significantly higher than that for CK. The 30 + 90 d-100 treatment resulted in the highest soluble sugar content, 60.5% higher than that for CK.

As shown in Table 2, exogenous MT treatment increased the sucrose content of pear fruit. Among them, the sucrose contents for the 30 d-100 and 30 + 90 d-100 treatments were significantly higher than that for CK at 11.0% and 9.4%, respectively. Sorbitol, fructose, and glucose contents increased after exogenous MT treatment. The sorbitol content for the 30 + 90 d-50 treatment was the highest at 4.193%, 20.8% higher than that for CK. The sorbitol contents for the 30 d-300 and 30 + 90 d-300 treatments decreased, but there was no significant difference compared with that for CK. The fructose content for 30 + 90 d-100 was the highest at 7.108%, 22.2% higher than that for CK. The glucose content for the 30 d-300 treatment was the highest at 1.537%, 83.4% higher than that for CK.

Table 3 shows that the malic acid content for the 30 + 90 d-100 treatment was significantly (18.6%) lower than that for CK, whereas those for other treatments were either higher than CK or not significantly different from CK. The citric acid content for each treatment with MT 90 d after full bloom was not significantly different from that for CK, whereas the citric acid contents for the 30 d-100, 30 d-300, 30 + 90 d-100, 30 + 90 d-200, and 30 + 90 d-300 treatments were significantly lower than that for CK. The quinic acid content of fruit treated at 90 d-50, 90 d-100, 90 d-200, 30 + 90 d-50, 30 + 90 d-200, and 30 + 90 d-300 decreased significantly. The quinic acid content for the 90 d-200 treatment was the lowest at 0.200 mg/g, 25.4% lower than that for CK, and the difference from the other treatments was not significant. The shikimic acid content of fruit treated with exogenous MT showed an increasing trend, and that for the 30 d-50 treatment was the highest at 0.083 mg/g, 62.7% higher than that for CK.

### 3.3. Effects of Exogenous MT on Texture Quality of Pear Fruit

**Figure 13:**
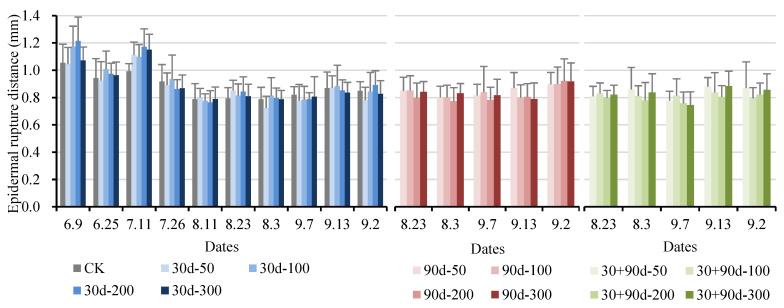
Changes in fruit epidermal rupture distance for different MT-spraying schemes.

The epidermal rupture distance refers to the puncture distance when the peel is ruptured after the fruit is punctured, which can indicate the toughness of the peel. As shown in Figure 13, in the early stage of fruit development, the epidermal rupture distance for the 30 d-100 and 30 d-200 treatments increased compared to that for CK. After 11 July, the epidermal rupture distance for each treatment was not significantly different (*p* > 0.05), showing no regular changes.

**Figure 14:**
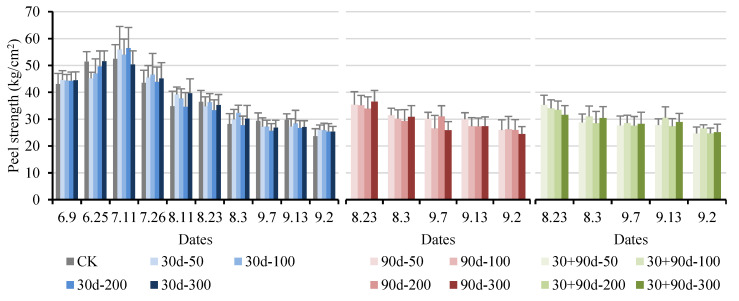
Changes in fruit peel strength for different MT-spraying schemes.

The peel puncture strength is the maximal force value before the peel ruptures during fruit puncture. The epidermal puncture strength of ‘Yuluxiang’ pears generally increased first and then decreased (Figure 14). The epidermal puncture strengths for the 90 d-50, 90 d-200, and 30 + 90 d-100 treatments were higher at the late stage of fruit development; however, from 7 September to 13 September, the epidermal puncture strength for the MT treatments showed a downward trend. At the fruit maturity stage, the epidermal puncture strength for the 30 + 90 d-100 treatment was 26.56 kg/cm^2^, significantly higher (12.4%) than that for CK.

**Figure 15:**
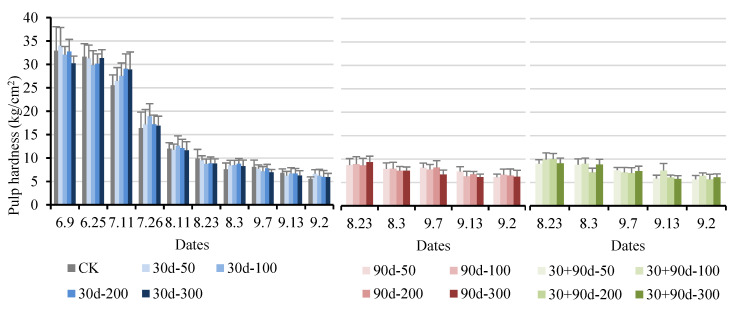
Firmness changes in pulp hardness for different MT-spraying schemes.

Figure 15 shows that the pulp hardness of ‘Yuluxiang’ pears gradually decreased with fruit ripening. The different MT treatments increased the average pulp hardness of pears to varying degrees, particularly during the mature stage. The average pulp hardness for the 90 d-200 treatment was the highest at 6.47 kg/cm^2^, 16.7% higher than that for CK. However, before fruit ripening, the pulp hardness after MT treatment showed a decreasing trend.

**Figure 16:**
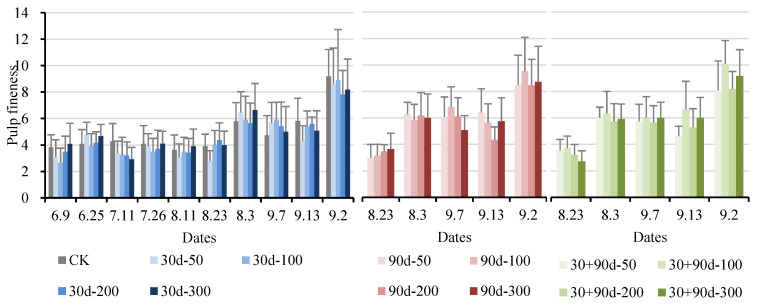
Changes in fruit pulp fineness for different MT-spraying schemes.

Pulp fineness refers to the fiber index of the pulp: the less fiber content in the pulp, the finer the pulp. Pulp fineness indicates the degree of pulp delicacy. The greater the fineness, the more delicate the pulp; the smaller the fineness, the rougher the pulp. The pulp fineness of ‘Yuluxiang’ pears gradually increased during fruit development. As shown in Figure 16, after 23 August, the pulp fineness for each treatment began to increase rapidly. After 13 September, the pulp fineness for the 30 + 90 d-100 treatment remained high, but the difference was not significant (*p* > 0.05).

**Figure 17:**
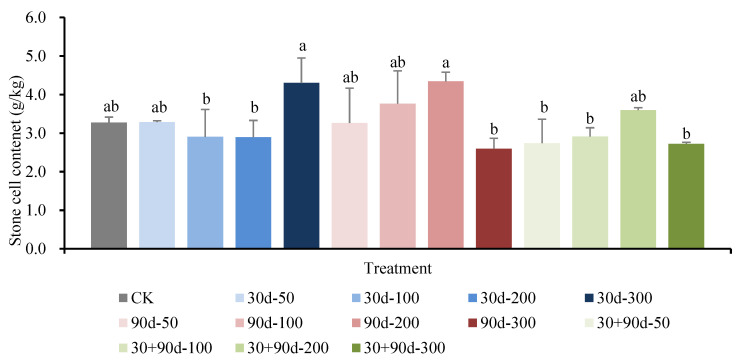
Changes in stone cell content of fruit in mature stage for different MT-spraying schemes. Different lowercase letters indicate that the content of the stone cell was significantly different in different treatments (*p* < 0.05).

As shown in Figure 17, the stone cell content of pear pulp in the different MT-spraying schemes was not significantly different from that of CK at the fruit maturity stage. Among them, the stone cell contents for the 30 d-300 and 90 d-200 treatments were higher, both approximately 4.3 g/kg.

### 3.4. Comprehensive Evaluation of MT-Spraying Schemes

To further understand the main factors affecting the quality of pear fruit using different MT-spraying schemes, 10 fruit quality index data points of longitudinal diameter, transverse diameter, single fruit weight, peel strength, pulp hardness, pulp fineness, soluble solid content, soluble sugar content, titratable acid content, and a* value were selected and analyzed using a PCA after min-max standardization. The Kaiser–Meyer–Olkin (KMO) test results showed a KMO value of 0.653 (greater than 0.6), indicating a correlation among the 10 indicators that met the PCA requirements. At the same time, through the Bartlett test, the P value was 0.005 (less than 0.05), showing a significance level of 1%, which can be used for a PCA (Table 4).

The total variance interpretation table examines the contribution rates of the principal components to variable interpretation (Table 5). In general, the higher the variance interpretation rate, the more important the principal component and the higher the weight ratio. When there were three principal components, the characteristic root of the total variance interpretation was greater than one, and the cumulative contribution rate of the variable interpretation reached 78.43, which determined the extraction of the first three principal components.

**Figure 18:**
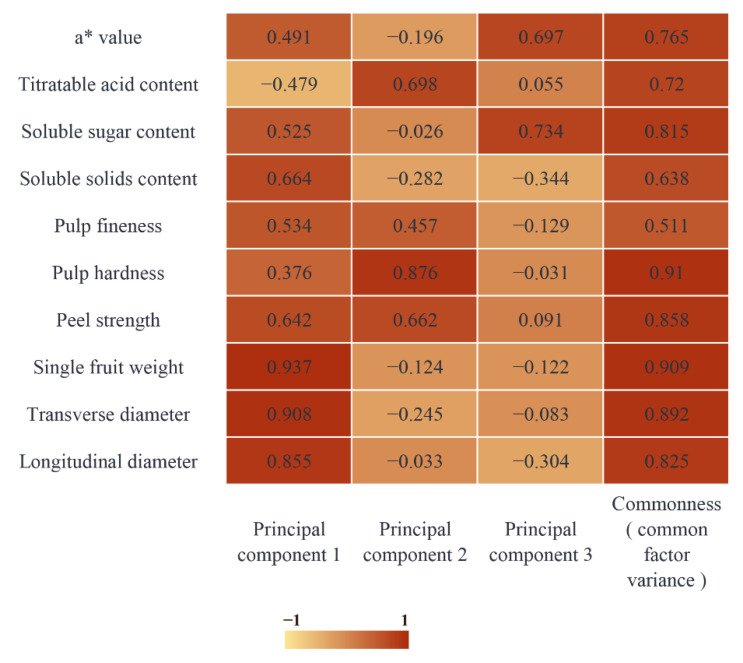
Thermal diagram of factor-load matrix.

The importance of the hidden variables in each principal component was analyzed using a factor-load matrix heat map (Figure 18). In principal component 1, the load coefficients of single fruit weight, transverse diameter, and longitudinal diameter were 0.937, 0.908, and 0.855, respectively, which were important factors in determining the variance of the first principal component. The important factors in principal component 2 were pulp hardness, titratable acid content, and peel strength, with load coefficients of 0.876, 0.698, and 0.662, respectively. The load coefficients of soluble sugar content and a* value in principal component 3 were 0.734 and 0.697, respectively.

**Figure 19:**
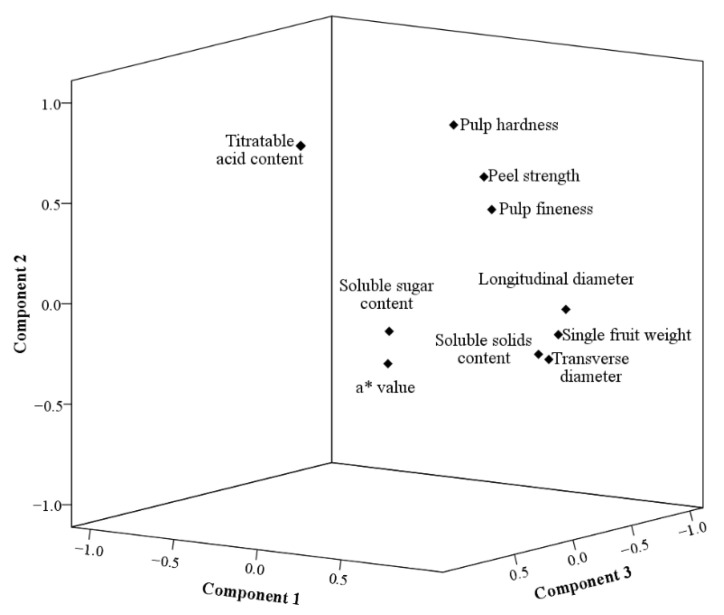
Factor-load quadrant analysis.

The factor-loading diagram reduced multiple factors into three principal components and presented the spatial distribution of the principal components through a quadrant diagram (Figure 19). A strong correlation was observed among pulp hardness, peel strength, and pulp fineness, as well as among longitudinal diameter, transverse diameter, single fruit weight, soluble solid content, soluble sugar, and a* value.

The weight score of each component factor (Table 6) was further calculated as linear combination coefficient × (variance interpretation rate/cumulative variance interpretation rate). Finally, it was normalized to the factor weight score. The linear combination coefficient formula was the factor load coefficient divided by the corresponding characteristic root. The formula for calculating each principal component score was then obtained:

F1 = 0.192 × longitudinal diameter + 0.204 × transverse diameter + 0.21 × single fruit weight + 0.144 × peel strength + 0.084 × pulp hardness + 0.12 × pulp fineness + 0.149 × soluble solids content + 0.118 × soluble sugar content − 0.107 × titratable acid content + 0.11 × a* value.

F2 = −0.016 × longitudinal diameter − 0.117 × transverse diameter − 0.059 × single fruit weight + 0.316 × peel strength + 0.418 pulp hardness + 0.218 × pulp fineness − 0.134 × soluble solids content − 0.012 × soluble sugar content + 0.333 × titratable acid content − 0.093 × a* value.

F3 = −0.237 × longitudinal diameter − 0.065 × transverse diameter − 0.095 × single fruit weight + 0.071 × peel strength − 0.024 × pulp hardness − 0.101 × pulp fineness − 0.268 × soluble solids content + 0.571 × soluble sugar content + 0.043 × titratable acid content + 0.542 × a* value.

The above fruit quality index values were standardized values.

According to information such as the load coefficient, a principal component weight analysis was conducted (Table 7). The results showed that the weight of principal component 1 was 56.882%, that of principal component 2 was 26.727%, and that of principal component 3 was 16.390%. The maximal value of the index weight was principal component 1, and the minimal value was principal component 3.

Finally, the comprehensive score for each MT-spraying scheme was calculated as F = α_1_F_1_ + α_2_F_2_ + α_3_F_3_. The higher the comprehensive score, the better the comprehensive fruit quality traits. As shown in Table 8, the comprehensive score of fruit quality for the 30 + 90 d-100 treatment was the highest (1.358), and the comprehensive score for CK was the lowest (−1.378).

## 4. Discussion

### 4.1. Exogenous MT Affects the Appearance Quality of Pear Fruit

Appearance quality is an important factor in determining the economic value of pears and consumer preferences. The size, shape, and color of fruit are important in judging their appearance. Many environmental factors affect the appearance quality of fruit, including light, water, temperature, etc. Supplementary light can increase the weight of the fruit and promote the coloring of the peel [34,35]. To ensure the normal development of plant fruit, ensuring normal water supply is necessary. Light and medium water stress from the young fruit stage to the mature stage reduces fruit weight [36]. High temperature can inhibit the biosynthesis of anthocyanins and stimulate peroxidase activity, thereby degrading anthocyanins in grape berries [37]. At the same time, some internal factors can also affect the appearance quality of fruit. Both auxin and gibberellic acid promote cell division and expansion, thereby regulating fruit development and enlargement [38]. Zhao et al. [39] observed a significant increase in MT concentration during cell elongation and expansion, suggesting MT involvement in these processes. This experiment showed that different MT-spraying concentrations and times at different stages could increase the vertical diameter, transverse diameter, and single fruit weight of pear fruit to varying degrees. Spraying 100 μmol/L MT twice at 30 and 90 d after full bloom had a better effect, which is consistent with the previous conclusions [6]. Similar results have been reported for peaches [40], apples [14], and blackberries [41]. MT and auxin have the same precursors and physiological functions; however, the relationship between them is unclear. However, studies in model plants showed that MT and auxin acted through different pathways to alter gene expression in *Arabidopsis thaliana* [42]. The relationship between MT and auxin in pear fruit requires further study. At the fruit maturity stage, the sun surface of ‘Yuluxiang’ pear fruit is be red, which greatly improves its appearance quality. Spraying 100 μmol/L MT promoted the accumulation of anthocyanins in grape skins, thereby promoting coloring [25]. Similar conclusions were drawn from experiments in this study. We found that spraying 100 μmol/L MT twice at 30 and 90 d after full bloom promoted peel redness.

### 4.2. Exogenous MT Affects the Internal Quality of Pear Fruit

The internal quality of fruit is one of the key concerns of consumers. It includes indicators of sugar, acid, vitamin C, and phenolics, of which the sugar–acid content is an important part of determining its flavor. The contribution of sugar–acid content to flavor depends not only on the levels of sugar and acid, but also on the types and relative proportions of sugar and acid. Most fruit ripening is characterized by sugar accumulation with acid decrease. Reasonable regulation of fruit sugar and acid contents is of great significance for improving fruit quality. Mineral element content can affect the sugar–acid content of fruits. Fruit nitrogen content was positively correlated with sugar composition and negatively correlated with organic acids [43]. Spraying calcium fertilizer could increase the sugar content of apples and reduce the acid content [44]. At the same time, hormones have a significant effect on the sugar–acid content of fruit. Abscisic acid could unload photosynthetic products from the phloem into developing fruit and enhanced sugar accumulation in apple vesicles [45]. Moreover, exogenous MT can affect the fruit inclusion content. Zhang et al. [46] found that exogenous MT treatment could increase the ratio of soluble solids to acids in fruit and upregulate the sucrose, glucose, fructose, and soluble sugar contents by regulating the activities and transcription levels of sucrose synthase, sucrose phosphate synthase, acid invertase, and neutral invertase. Wu et al. [40] showed that exogenous MT treatment increased the content of soluble solids, fructose, sorbitol, and sucrose in peaches and decreased the content of glucose. Du et al. [47] found that MT application increased the soluble sugar content of tomato fruit and reduced the organic acid content. Liu et al. [6] treated ‘Zaosu’ pear trees with MT and found that the soluble sugar content in the fruit increased, among which the sucrose and sorbitol contents increased significantly. This study found that exogenous MT treatment increased the soluble solids and soluble sugar content of pear fruit and reduced the titratable acid content. Among them, spraying once only at 30 d after full bloom and spraying once only at 90 d after full bloom with a high concentration (300 μmol/L) of MT increased the titratable acid content in the pear fruit. We measured the sugar and acid components of each treatment at the mature stage and found that exogenous MT treatment significantly reduced the content of malic acid and citric acid in pear fruit and increased the content of sucrose, sorbitol, fructose, glucose, and shikimic acid. This is different from the above research conclusions, which may be caused by the different tree species and varieties. In addition, rainfall, light, and the proportion and content of soil mineral elements may have different effects on the sugar and acid content of fruit at the mature stage.

### 4.3. Exogenous MT Affects the Texture of Pear Fruit

Fruit texture is closely related to the mechanical strength of cell walls and intercellular swelling pressure [48]. Peel toughness, peel strength, pulp hardness, and pulp fineness directly affect the taste of fruit, their transportation, and their storage. Some plant hormones can affect fruit texture. Exogenous ethylene treatment promoted cell wall degradation by stimulating relevant enzyme activities and gene expression, thereby promoting softening in blueberries [49]. Abscisic acid had a similar function [50]. The peel of ‘Yuluxiang’ pears is thin, and the pulp is delicate. Fruits are susceptible to mechanical damage during picking, transportation, and storage, resulting in economic losses. Improving the above problems through economically feasible cultivation measures can greatly improve production efficiency. This experiment showed that exogenous MT treatment could increase peel strength and pulp hardness at the fruit-ripening stage; however, peel strength and pulp hardness showed a decreasing trend before fruit ripening, which may explain why MT stimulates fruit ripening by inducing ethylene synthesis [14].

### 4.4. Screening of Exogenous MT-Spraying Schemes

A PCA can transform a large number of related indicators into a small number of comprehensive indicators with low correlations [51]. This method can be used to calculate the comprehensive score of each treatment, while reducing information loss. Peng et al. [52] comprehensively evaluated 15 quality indices of kiwifruit after different MT treatments using a PCA, screened four principal components, and determined that 300 μmol/L MT treatment had the greatest effect on enhancing disease resistance and maintaining fruit quality of kiwifruit. A PCA of 10 indices of 20 peach varieties confirmed that the chemical composition of peach fruit varied significantly among the varieties [53]. The results of the PCA in this study showed that the cumulative contribution rate of the first three principal component variables reached 78.43%. The first principal component was determined using single fruit weight, transverse diameter, and longitudinal diameter. The second principal component was determined based on pulp hardness, titratable acid content, and peel strength. The third principal component was determined via analyzing the soluble sugar content and red–green value. The effect of each MT-spraying scheme on the improvement of pear fruit quality was determined based on the score ranking, and the optimal MT-spraying scheme was selected. The results of this experiment showed that the comprehensive score of 100 μmol/L MT sprayed twice at 30 and 90 d after full bloom was the highest, and the effect on improving pear fruit quality was better.

## 5. Conclusions

The results of this study demonstrated that preharvest MT spraying significantly affected pear fruit quality. Spraying 100 μmol/L MT twice at 30 and 90 d after full bloom had the best effect and significantly improved the appearance quality of pear fruit (including weight, size, and color), increased the sugar content of the fruit, reduced the acid content, and improved the peel strength. These findings present a fresh approach to improving pear quality and provide a theoretical basis for the application of MT in fruit production.

## Figures and Tables

**Table 1:** MT-spraying schemes.

Order Number	Treatment	Spraying Time	Spray Concentration (in μmol/L)	Spraying Times (No. of Times)
1	30 d-50	30 d after full bloom	50	one
2	30 d-100	30 d after full bloom	100	one
3	30 d-200	30 d after full bloom	200	one
4	30 d-300	30 d after full bloom	300	one
5	90 d-50	90 d after full bloom	50	one
6	90 d-100	90 d after full bloom	100	one
7	90 d-200	90 d after full bloom	200	one
8	90 d-300	90 d after full bloom	300	one
9	30 + 90 d-50	30 + 90 d after full bloom	50	two
10	30 + 90 d-100	30 + 90 d after full bloom	100	two
11	30 + 90 d-200	30 + 90 d after full bloom	200	two
12	30 + 90 d-300	30 + 90 d after full bloom	300	two

**Table 2:** Contents of sugar components in fruit for different MT-spraying schemes at maturity stage.

Treatment	Sugar Components (%)			
	Sucrose	Sorbitol	Fructose	Glucose
CK	2.721 ± 0.067 b	3.470 ± 0.029 f	5.819 ± 0.227 d	0.838 ± 0.071 g
30 d-50	1.636 ± 0.023 g	3.708 ± 0.012 de	7.079 ± 0.208 a	1.145 ± 0.034 cd
30 d-100	3.020 ± 0.045 a	4.168 ± 0.023 ab	5.880 ± 0.234 cd	1.076 ± 0.005 de
30 d-200	2.431 ± 0.011 c	4.036 ± 0.132 abc	5.945 ± 0.162 cd	1.024 ± 0.039 ef
30 d-300	1.171 ± 0.023 h	3.450 ± 0.014 f	6.692 ± 0.188 b	1.537 ± 0.119 a
90 d-50	1.778 ± 0.010 f	3.673 ± 0.027 def	6.645 ± 0.029 b	1.213 ± 0.003 bc
90 d-100	2.477 ± 0.034 c	3.602 ± 0.120 ef	6.171 ± 0.240 c	0.979 ± 0.043 ef
90 d-200	1.947 ± 0.094 e	4.072 ± 0.093 abc	6.867 ± 0.193 ab	1.270 ± 0.057 b
90 d-300	2.231 ± 0.043 d	3.860 ± 0.127 cd	6.125 ± 0.001 cd	0.949 ± 0.033 f
30 + 90 d-50	2.495 ± 0.026 c	4.193 ± 0.034 a	6.705 ± 0.332 b	1.124 ± 0.050 cd
30 + 90 d-100	2.977 ± 0.061 a	4.165 ± 0.341 ab	7.108 ± 0.092 a	1.143 ± 0.067 cd
30 + 90 d-200	1.601 ± 0.018 g	3.941 ± 0.080 bc	6.923 ± 0.250 ab	1.258 ± 0.082 b
30 + 90 d-300	1.754 ± 0.040 f	3.446 ± 0.169 f	6.663 ± 0.113 b	1.215 ± 0.037 bc

Different lowercase letters indicate that the content of the same component was significantly different in different treatments (*p* < 0.05).

**Table 3:** Content of acid components in fruit for different MT-spraying schemes at maturity stage.

Treatment	Acid Components (mg/g)			
	Malic Acid	Citric Acid	Quinic Acid	Shikimic Acid
CK	3.114 ± 0.048 gh	0.146 ± 0.011 bc	0.268 ± 0.008 ab	0.051 ± 0.003 h
30 d-50	3.160 ± 0.101 fgh	0.180 ± 0.002 a	0.281 ± 0.022 a	0.083 ± 0.004 a
30 d-100	3.258 ± 0.017 de	0.125 ± 0.001 d	0.256 ± 0.003 bc	0.059 ± 0.002 ef
30 d-200	3.082 ± 0.010 h	0.157 ± 0.006 b	0.258 ± 0.008 bc	0.057 ± 0.002 fg
30 d-300	2.971 ± 0.021 i	0.115 ± 0.005 de	0.256 ± 0.009 bc	0.071 ± 0.002 b
90 d-50	3.607 ± 0.037 a	0.158 ± 0.011 b	0.203 ± 0.005 f	0.060 ± 0.001 def
90 d-100	3.424 ± 0.015 c	0.148 ± 0.011 bc	0.227 ± 0.002 de	0.062 ± 0.006 cdef
90 d-200	3.207 ± 0.047 def	0.145 ± 0.001 c	0.200 ± 0.005 f	0.054 ± 0.002 gh
90 d-300	3.275 ± 0.047 d	0.147 ± 0.002 bc	0.271 ± 0.017 ab	0.061 ± 0.002 def
30 + 90 d-50	3.511 ± 0.076 b	0.185 ± 0.002 a	0.243 ± 0.012 cd	0.054 ± 0.003 gh
30 + 90 d-100	2.535 ± 0.015 j	0.060 ± 0.004 f	0.278 ± 0.007 a	0.065 ± 0.002 cd
30 + 90 d-200	3.179 ± 0.110 efg	0.105 ± 0.007 e	0.225 ± 0.005 e	0.067 ± 0.002 bc
30 + 90 d-300	3.251 ± 0.040 def	0.112 ± 0.004 e	0.243 ± 0.006 g	0.064 ± 0.002 cde

Different lowercase letters indicate that the content of the same component was significantly different in different treatments (*p* < 0.05).

**Table 4:** KMO inspection and Bartlett inspection.

Test		Results
KMO value		0.653
Bartlett sphericity test	Approximate chi-squared	76.137
	df	45
	P	0.005 ***

*** represent the significance levels of 1%.

**Table 5:** Variance interpretation table.

Components	Latent Root	Variance Explained Rate (in %)	Cumulative Variance Interpretation Rate (in %)
1	4.461	44.613	44.613
2	2.096	20.962	65.576
3	1.285	12.855	78.43
4	0.732	7.317	85.748
5	0.565	5.651	91.399
6	0.374	3.738	95.137
7	0.297	2.975	98.112
8	0.1	0.999	99.111
9	0.074	0.743	99.854
10	0.015	0.146	100

**Table 6:** Weight score of each component factor.

Index	Component 1	Component 2	Component 3
Longitudinal diameter	0.192	−0.016	−0.237
Transverse diameter	0.204	−0.117	−0.065
Single fruit weight	0.210	−0.059	−0.095
Peel strength	0.144	0.316	0.071
Pulp hardness	0.084	0.418	−0.024
Pulp fineness	0.120	0.218	−0.101
Soluble solids content	0.149	−0.134	−0.268
Soluble sugar content	0.118	−0.012	0.571
Titratable acid content	−0.107	0.333	0.043
a* value	0.110	−0.093	0.542

**Table 7:** Factor weight analysis.

Component	Variance Explained Rate (%)	Cumulative Variance Interpretation Rate (%)	Weight (%)
Component 1	44.613	44.613	56.882
Component 2	20.962	65.576	26.727
Component 3	12.855	78.430	16.390

**Table 8:** Comprehensive score of each MT-spraying scheme.

Ranking	Treatment	Comprehensive Score	Component 1	Component 2	Component 3
1	30 + 90 d-100	1.358	1.863	0.556	0.916
2	90 d-200	0.647	0.996	0.545	−0.398
3	30 d-100	0.495	0.661	0.131	0.511
4	90 d-100	0.42	0.141	1.381	−0.18
5	90 d-50	0.137	0.221	0.328	−0.469
6	30 + 90 d-300	−0.081	0.334	−0.335	−1.108
7	30 + 90 d-200	−0.171	0.907	−2.146	−0.69
8	30 d-50	−0.205	−0.746	1.297	−0.776
9	30 d-200	−0.246	−0.056	−0.346	−0.744
10	30 d-300	−0.292	−1.079	0.319	1.442
11	90 d-300	−0.302	−0.986	0.37	0.976
12	30 + 90 d-50	−0.382	−0.473	−1.498	1.753
13	CK	−1.378	−1.783	−0.602	−1.235

## Data Availability

The datasets generated and analyzed during the current study are available from the corresponding author upon reasonable request.
